# Brain-enriched microRNAs circulating in plasma as novel biomarkers for Rett syndrome

**DOI:** 10.1371/journal.pone.0218623

**Published:** 2019-07-10

**Authors:** Kira Sheinerman, Aleksandra Djukic, Vladimir G. Tsivinsky, Samuil R. Umansky

**Affiliations:** 1 DiamiR, LLC, Princeton, NJ, United States of America; 2 Albert Einstein College of Medicine, Montefiore Medical Center, New York, NY United States of America; National Institutes of Health, UNITED STATES

## Abstract

Rett syndrome (RTT) is a severe neurodevelopmental disorder caused by mutations in the X-linked gene *MECP2* (methyl-CpG-binding protein 2). Minimally invasive and accurate biomarkers of disease progression and treatment response could facilitate screening of therapeutic compounds in animal models, enrollment of better-defined participants into clinical trials, and treatment monitoring. In this study, we used a targeted approach based on analysis of brain-enriched microRNAs (miRNAs) circulating in plasma to identify miRNA biomarkers of RTT using *Mecp2*-mutant mice as a model system and human plasma samples. An “miRNA pair” approach, i.e. the ratio between two miRNAs, was used for data normalization. Specific miRNA pairs and their combinations (classifiers) analyzed in plasma differentiated wild-type from *Mecp2* male and female mice with >90% accuracy. Individual miRNA pairs were more effective in distinguishing male (homozygous) animals than female (heterozygous) animals, suggesting that disease severity correlated with the levels of the miRNA biomarkers. In the human study, 30 RTT patients were compared with age-matched controls. The results of this study showed that miRNA classifiers were able to differentiate RTT patients from controls with 85–100% sensitivity. In addition, a comparison of various age groups demonstrated that the dynamics in levels of miRNAs appear to be associated with disease development (involvement of liver, muscle and lipid metabolism in the pathology). Importantly, certain miRNA biomarker pairs were common to both the animal models and human subjects, indicating the similarity between the underlying pathological processes. The data generated in this feasibility study suggest that circulating miRNAs have the potential to be developed as markers of RTT progression and treatment response. Larger clinical studies are needed to further evaluate the findings presented here.

## Introduction

Rett syndrome (RTT) is a neurodevelopmental disorder caused by mutations in the *MECP2* gene, which encodes the transcriptional regulatory protein methyl-CpG-binding protein-2 (MECP2) [1–3]. RTT has an X-linked dominant inheritance pattern, with most mutations arising spontaneously in the paternal germ line. Because hemizygosity in males is usually lethal in the perinatal period, the vast majority of RTT patients are heterozygous females. Affected females appear normal at birth, and disease onset is typically evident by 6–18 months of age and is characterized by neurological regression, microcephaly, loss of acquired speech and motor stereotypies [4–7]. Clinical severity appears to depend on multiple factors, including the type of *MECP2* mutation, skewing of the X-inactivation and likely genetic modifiers of *MECP2*. Although gross brain cytoarchitecture is normal in this condition, and RTT does not cause excessive neuronal cell death or degeneration, there is considerable evidence that loss of MECP2 leads to widespread disruption of microcircuit structure and function throughout the neuraxis [8–13]. Numerous mouse models of RTT have been generated and are widely used to study brain pathologies associated with the loss of MECP2 and for preclinical drug development [14–18]. Although diagnostic *MECP2* genetic testing is available for RTT, biomarkers of RTT, including blood-borne indicators of disease severity and progression, are lacking.

Recently, we demonstrated the potential use of circulating microRNAs (miRNAs) as biomarkers for early detection of Alzheimer’s (AD) and other neurodegenerative diseases (NDs) [19–21]. miRNAs are comprised of small, single-stranded, highly conserved, non-coding RNA sequences that are approximately 22 nucleotides long [22] and that play critical roles in post-transcriptional regulation of gene expression [23]. We hypothesized that the synaptic and neuritic dysfunction and destruction common among numerous neurodegenerative diseases would result in different levels of miRNA secretion and excretion in affected brain regions, with subsequent changes in brain-enriched miRNA plasma concentrations [20, 23]. To test this hypothesis, plasma levels of circulating brain-enriched miRNAs present in the synapses of brain regions affected by a particular disease (e.g., the hippocampus in early-stage AD or the midbrain and frontal cortex in Parkinson’s disease) were analyzed [21]. To control for the possible effects of unrelated variables that may alter synaptic miRNA levels in plasma (e.g., changes in blood supply or blood-brain barrier permeability), plasma concentrations of miRNAs enriched in other brain regions or cell types were measured simultaneously and were used as normalizers. Thus, in this approach, an effective biomarker is represented by the ratio of miRNAs present in synapses and enriched in brain regions affected by pathology (numerator) to miRNAs enriched in other brain regions (denominator). The resulting miRNA ratios (pairs) have been found to effectively distinguish individuals with mild cognitive impairment, and even pre-symptomatic AD, from age-matched controls and to detect circulating miRNA patterns characteristic of normal brain aging [20, 21, 23].

Although there is no evidence of neuronal degeneration in RTT, loss of MECP2 results in synaptic and neuritic dysfunction and marked dysregulation of miRNA species in the brain [9, 24–28]. Changes in miRNA expression patterns have been reported in the brain in mouse models of RTT and other neurodevelopmental disorders [29–31] and in bodily fluids of children with autism spectrum disorder [32, 33]. Given that brain-enriched miRNAs can be detected in the blood, these observations suggest that circulating miRNAs may be of value in monitoring disease severity, progression and treatment response in RTT. In addition, analysis of miRNAs enriched in other organs and of the metabolic processes affected by RTT may be useful for early detection and monitoring [34]. miRNA nucleotide sequences are highly conserved across species, and thus, miRNA pairs are likely to demonstrate utility as biomarkers in both murine and human RTT applications. In the current study, we used four murine RTT models (see below) and analyzed eight miRNAs pre-selected on the basis of their enrichment in the brain. Then, plasma from human patients was tested for 19 pre-selected miRNAs. Additionally, since RTT development occurs at a young age, which is a time when various parameters within the body are undergoing changes, the data from the RTT patients were compared with data from age-matched controls (AMCs).

## Results

### RTT mouse models study

#### miRNA selection

Table 1 lists the miRNAs used in the present study. Because loss of MECP2 affects neurons throughout the neuraxis, miRNAs that have been shown to be enriched in synapses of both forebrain and brainstem regions were used, including miR-107, miR-132, miR-411 and miR-491-5p [35–40]. Additional miRNAs enriched in several brain regions (miR-323-3p) or predominantly in the pituitary gland (miR-335-5p, miR-370), as well as miR-16, a ubiquitously expressed miRNA involved in apoptosis regulation, were also analyzed. Predictive analysis indicates that some of the selected miRNAs may affect expression of RTT-related proteins (Table 1).

**Table 1:** miRNAs used in the study.

#	miRNA	Brain enrichment	Enriched in synapses	Mouse models study	Human RTT study	Down-regulated RTT-related proteins [41–44]*
1	**Let-7b-5p**	Ubiquitous			+	MECP2, TBL1, TUBA1B
2	**miR-16**	Ubiquitous		+	+	NCOR1, SIRT, CREB, BDNF
3	**miR-29b-3p**	PG (not brain-enriched)			+	
4	**miR-107**	FC, PG, Hip, MB	+	+	+	TUBA1B, CREB
5	**miR-122**	Liver-enriched			+	
6	**miR-125b**	FC, MB, PG, Hip	+		+	NCOR1, SIRT
7	**miR-132-3p**	PG, Hip	+	+	+	MECP2, SIRT, S100, BDNF
8	**miR-134**	MB, Hip, PG	+		+	CREB, BDNF
9	**miR-146a**	Inflammatory			+	S100
10	**miR-155**	Inflammatory			+	MECP2, S100
11	**miR-181a-5p**	MB, FC	+		+	MECP2, TBL1, S100, NREP, CREB, BDNF
12	**miR-206**	Muscle, Cer			+	
13	**miR-323-3p**	FC, Hip, MB	+	+	+	
14	**miR-335-5p**	PG, Hip		+	+	TBL1, GFAP, S100, CREB
15	**miR-370**	PG, FC	+	+	-	
16	**miR-409-3p**	Hip	+		+	NR2F2
17	**miR-411-5p**	PG, Hip, FC		+	+	
18	**miR-432-5p**	PG, MB, Cer			+	MECP2
19	**miR-433-3p**	PG, MB	+		+	CREB
20	**miR-491-5p**	MB, FC	+	+	+	

Cer–Cerebellum; FC–Frontal Cortex; Hip–Hippocampus; MB–Midbrain; PG–Pituitary Gland. “+” indicates miRNAs enriched in synapses and miRNAs tested in the mouse models and human RTT studies

*http://mirtarbase.mbc.nctu.edu.tw/php/search.php

#### Male RTT model

**Fig 1:**
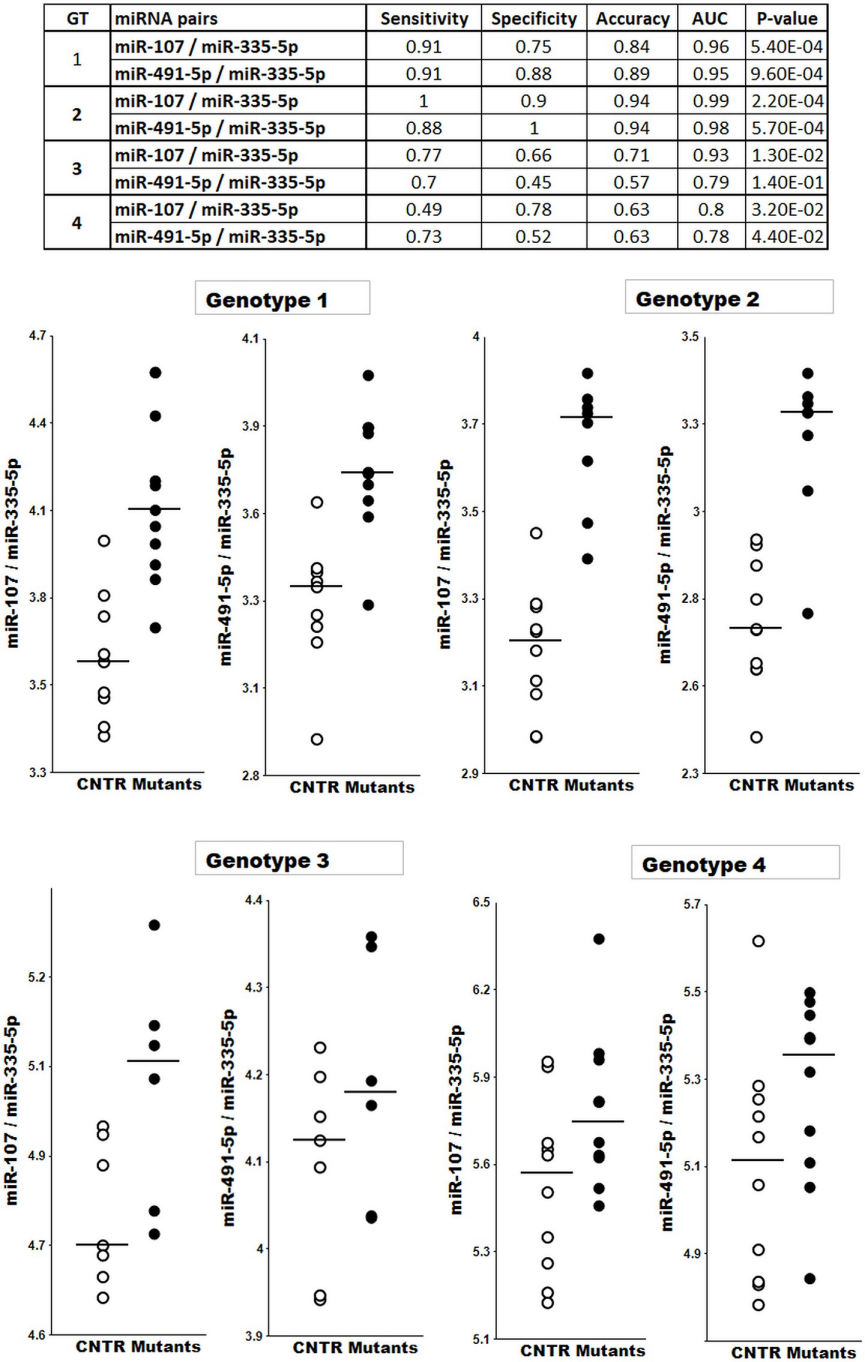
Differentiation between four Mutant and Control mice genotypes (GT). Genotypes: 1—male, *Mecp2*^*tm1*.*1Jae*^ Null; 2—male, *Mecp2*^***tm1*.*1Bird***^ Null; 3 –female, *Mecp2*^*tm1*.*1Jae*^ Het; 4—female, *Mecp2*^*tm1*.*1Bird*^ Het; CNTR–Control.

In the first experiment, the levels of eight miRNAs were measured in plasma samples from 11 *Mecp2*^*tm1*.*1Jae*^ Null and 9 wild-type (Wt) male mice aged 5–6 weeks. One control sample did not meet the quality control parameters and was thus excluded from the analysis. The dot plots, receiver operator characteristic (ROC) curves, sensitivity, specificity and accuracy for the resulting miRNA pairs are presented in Fig 1 and S1 Fig. Several miRNA pairs and their combinations distinguished Wt from *Mecp2* ^*tm1*.*1Jae*^ mice with 89–95% accuracy. These results were encouraging, particularly because two effective numerators in the miRNA pairs, namely, miR-107 and miR-491-5p, are expressed in synapses of the forebrain and brainstem.

To confirm the data obtained in the pilot experiment, we repeated the experiments with 8 *Mecp2*^*tm1*.*1Bird*^ Null and 10 Wt male mice aged 5–6 weeks. The same eight miRNAs were analyzed in this cohort of plasma samples. Fig 1 presents the data obtained with the same miRNA pairs first identified in the initial experiment with *Mecp2*^*tm1*.*1Jae*^ mice. The results obtained from both mouse strains were very similar. However, in the *Mecp2*^*tm1*.*1Bird*^ animals, additional miRNA pairs were identified that could effectively differentiate between Wt and *Mecp2* Null mice (S1 Fig).

Although the sample sizes in both the pilot study and the confirmatory study were relatively small (n = 19 and n = 18 samples, respectively), the reproducibility of the findings from these two different RTT mouse models obtained by different labs strengthened the conclusion that plasma levels of the selected brain-enriched miRNAs can reliably distinguish between Wt and Null animals.

#### Female RTT model

We then compared the levels of the eight miRNAs in two cohorts of female heterozygous (Het) and Wt mice: *Mecp2*^*tm1*.*1Jae*^ Het n = 6, Wt n = 7 (6 months old); and *Mecp2*^*tm1*.*1Bird*^ Het n = 10, Wt n = 10 (18 weeks old). The two pairs of mutant and Wt cohorts were tested at different ages and, therefore, were analyzed independently. Fig 1 and S2 Fig present the data from these two experiments as dot plots, ROC curves, sensitivity, specificity and accuracy. As expected, in the Het mice, the area under the ROC curve (AUC) and associated accuracy, which represented the ability of miRNA pairs to distinguish mutant from Wt mice, were lower than those in the Null mice since female heterozygotes retain some normal *Mecp2* expression. However, classifiers consisting of combined miRNA pairs provided >90% of the overall accuracy in the female Het *Mecp2*^*tm1*.*1Jae*^ and *Mecp2*^*tm1*.*1Bird*^ mouse models. The AUC values for all four experiments are summarized in Table 2. Notably, certain miRNA pairs effectively distinguished between mutant and Wt mice consistently across all four animal models (*Mecp2*^*tm1*.*1Jae*^ Null and Het and *Mecp2*^*tm1*.*1Bird*^ Null and Het).

**Table 2:** Summary (mouse models and human RTT studies) of the areas under the ROC curves (AUC).

miRNA pairs	Mouse models study				Human RTT study		
	1	2	3	4	2–5 y.o.	6–15 y.o.	>15 y.o.
	n = 19	n = 18	n = 13	n = 20	n = 17	n = 24	n = 14
**miR-107 / miR-323-3p**	0.96	0.82	0.79	0.8	0.76		
**miR-107 / miR-335-5p**	0.96	0.99	0.93	0.8	0.72	0.84	
**miR-107 / miR-411-5p**	0.92		0.75				
**miR-107 / miR-132-3p**	0.86	0.98	0.71	0.82	0.85	0.65	
**miR-107 / miR-16**	0.83		0.71		0.88		
**miR-107 / miR-491-5p**	0.73		0.8		0.70	0.67	0.91
**miR-491-5p / miR-323-3p**	0.97	0.81		0.77	0.65		
**miR-491-5p / miR-335-5p**	0.96	0.98	0.79	0.78		0.81	
**miR-491-5p / miR-370**	0.89						
**miR-491-5p / miR-411-5p**	0.87		0.7				
**miR-491-5p / miR-132-3p**	0.87	0.99		0.82	0.90	0.67	
**miR-491-5p / miR-16**	0.97	0.75		0.71	0.87		
**miR-16 / miR-323-3p**	0.88	0.79	0.73	0.75			0.72
**miR-16 / miR-335-5p**	0.91	0.98	0.95	0.75			0.80
**miR-16 / miR-411-5p**	0.76		0.79				0.77
**miR-16 / miR-132-3p**	0.7	1					0.70
**miR-132-3p / miR-323-3p**	0.82		0.81				
**miR-132-3p / miR-335-5p**	0.84	0.89	0.96				0.91
**miR-411-5p / miR-323-3p**	0.77	0.84			0.88	0.71	
**miR-411-5p / miR-132-3p**		0.93			0.81	0.65	

Genotypes: 1—male, *Mecp2*^*tm1*.*1Jae*^ Null; 2—male, *Mecp2*^***tm1*.*1Bird***^ Null; 3 –female, *Mecp2*^*tm1*.*1Jae*^ Het; 4—female, *Mecp2*^*tm1*.*1Bird*^ Het.

### Human RTT study

**Fig 2:**
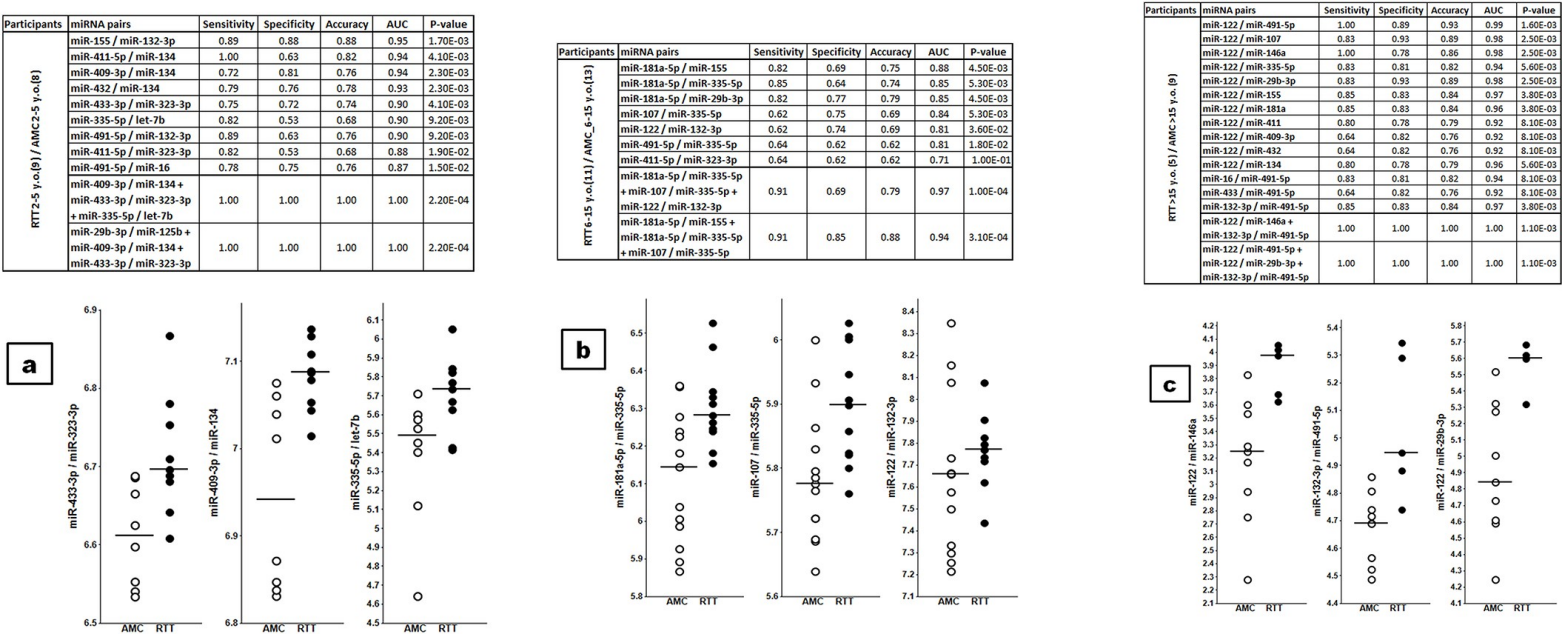
(a) Differentiation of the 2–5 y.o. RTT age group from the 2–5 y.o. AMC group. (b) Differentiation of the 6–15 y.o. RTT age group from the 6–15 y.o. AMC group. (c) Differentiation of the >15 y.o. RTT age group from the >15 y.o. AMC group.

**Fig 3:**
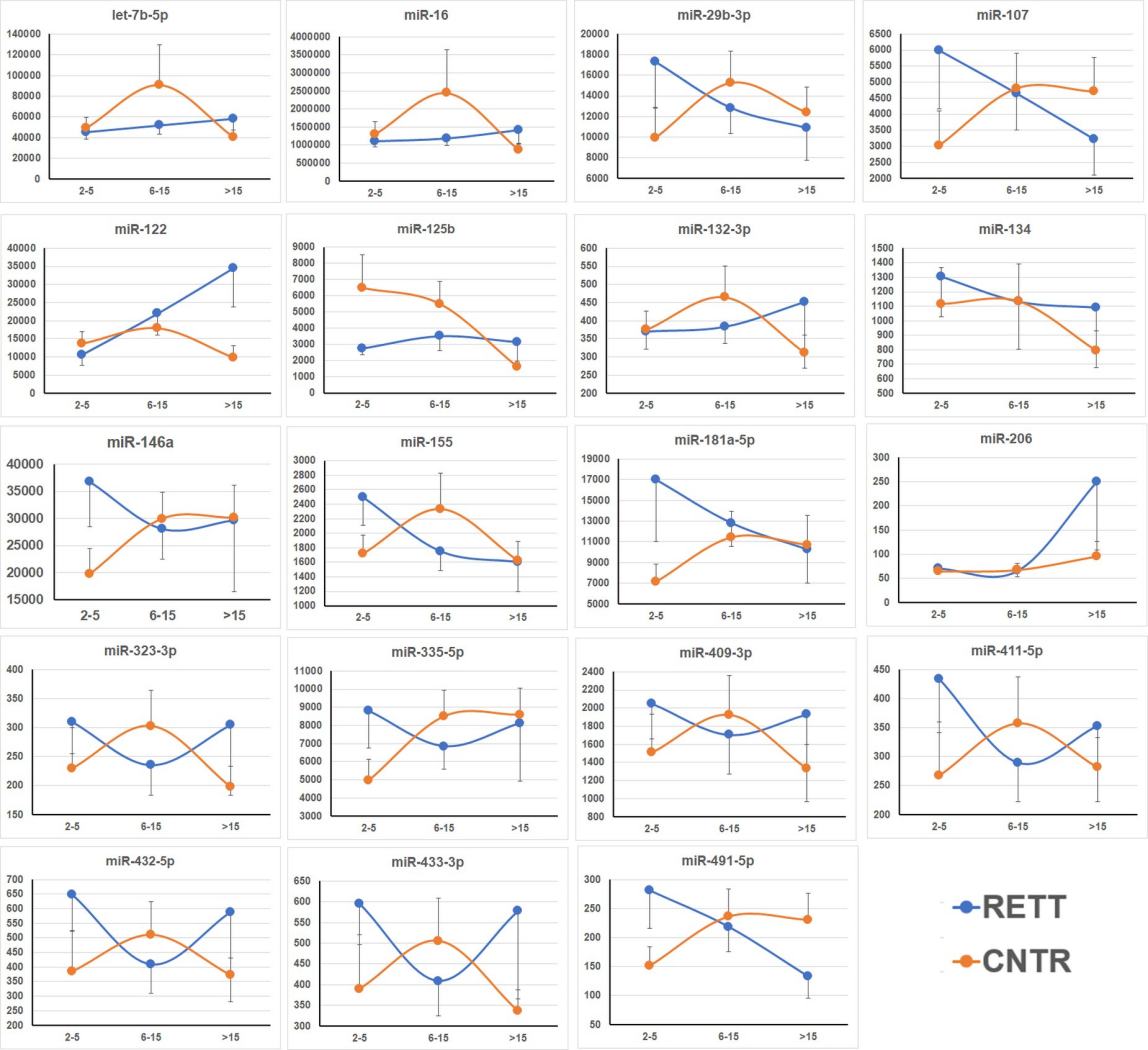
Age-dependent changes in plasma concentrations of the miRNAs in RTT patients (RETT) and AMCs (CNTR). The X and Y axes indicate age and the number of miRNA copies per 1 μl of plasma, respectively.

Thirty RTT patients and 30 female age-matched control (AMC) participants were enrolled in the study (Table 3 and S1 Table). Since larger volumes of human plasma were available, 19 miRNAs (Table 1) were tested, 7 of which were identical to those tested in the mouse model studies, and 4 were additional miRNAs that were shown to inhibit MECP2 synthesis (http://mirtarbase.mbc.nctu.edu.tw/php/search.php). Further, inflammatory miR-146a and miR-155, liver-enriched miR-122, and muscle-enriched miR-206, as well as additional brain-enriched miRNAs were tested (Table 1). RTT patient groups stratified according to age, namely, 2–5 years old, 6–15 years old, and >15 years old, were effectively differentiated from the AMCs by miRNA pairs and classifiers with an accuracy up to 100%, 94–97%, and 100%, respectively (Fig 2A–2C). Moreover, disease progression was reflected by changes in levels of miRNAs enriched in organs involved in a specific pathology. For example, the appearance of miR-122 as the numerator reflected the development of liver pathology. Table 2 demonstrates that certain miRNA pairs were common among different animal RTT models or human RTT in all, or at least some, age cohorts. The pathological progression was additionally demonstrated by the differentiation of the RTT groups from each other (S3 Fig). miR-122, as a biomarker of liver pathology, was the numerator that performed best in separating RTT groups of various ages. Fig 3 demonstrates that the dynamics of age-dependent changes in the plasma levels of the tested miRNAs differed substantially between the RTT and AMC groups. S2 Table provides a summary of miRNA pairs that effectively differentiated RTT age groups but did not differentiate the respective AMC groups and vice versa.

**Table 3:** Demographics and clinical data of RTT patients.

#	Age at BD	Genetics	Phenotype	Seizures	Ambulatory	Breathing dysfunction	Cholesterol (mg/dL)	ALT liver enzymes (U/L)
1	2	T241K	classical rett	no	WCB/NA	none	144 (76–216)	15 (0–20)
2	2	C312G>T	classical rett	no	WCB/NA	none	182 (108–193)	30 (0–20)
3	3	R168X	classical rett	yes	AMB	HV	190 (108–193)	13 (< = 25)
4	3	T158M	classical rett	yes	WCB/NA	HV, BH	199 (108–193)	9 (0–20)
5	3	G238fs	classical rett	no	WCB/NA	none	154 (76–216)	16 (0–20)
6	4	C502T	classical rett	no	AMB	irregular only when waking up	118 (108–193)	14 (0–20)
7	5	deletion in exon 4	classical rett	yes	AMB	none	203 (106–193)	13 (0–20) L
8	5	deletion	classical rett	no	WCB/NA	HV, BH	230 (106–193)	11(0–20)
9	5	T158M	classical rett	no	AMB	HV	99 (106–193)	17 (0–20)
10	6	T158M	classical rett	yes	AMB	BH	98 (106–198)	14 (0–20)
11	7	R270X	classical rett	no	AMB	HV	154 (106–193)	20 (10–35)
12	8	R270X	classical rett	no	AMB	HV	141 (105–218)	17 (0–20)
13	8	P152A	classical rett	no	AMB	HV	143 (105–218)	19 (0–20)
14	9	deletion	classical rett	no	AMB	MBH	156 (105–218) L	14 (< = 25)
15	9	R255X	classical rett	no	AMB	none	116 (108–170)	20 (< = 25)
16	10	R168X	classical rett	yes	WCB/NA	apnea, HV, BH	143 (105–218)	26 (0–20)
17	10	deletion	HF	no	AMB	none	89 (105–218)	9 (0–20)
18	13	R270X	classical rett	yes	WCB/NA	BH	171 (108–170)	15 (0–20)
19	13	deletion	classical rett	no	AMB	BH	111 (105–218)	23 (0–20)
20	14	T158M	classical rett	yes	AMB	BH	133 (108–170)	29 (< = 25)
21	15	PSNA	HF	no	AMB	none	144 (108–170)	12 (0–20)
22	15	R306C	classical rett	yes	AMB	BH	146 (<170)	23 (0–31)
23	15	C916C	HF	no	AMB	MBH	175 (108–170)	8 (0–20)
24	18	T158M	classical rett	yes	AMB	BH	125 (110–170)	18 (0–20)
25	19	R270X	classical rett	yes	WCB/NA	none	127 (<200)	60 (9–32)
26	21	R133C	classical rett	yes	AMB	MBH	147 (122–200)	10 (0–20)
27	23	Xq28	HF	no	AMB	BH	104 (<200)	29 (5–45)
28	32	L386fs	classical rett	no	WCB/NA	none	166 (100–199)	38 (0–32)
29	33	PSNA	classical rett	yes	WCB/NA	HV	217 (128–200)	9 0–20)
30	34	P225R	HF	no	AMB	MBH	135 (125–200)	28 (6–29)

BD–blood draw; PSNA—positive, specifies not available; HF—high functioning; WCB/NA–wheelchair bound / non-ambulatory; AMB—ambulatory; HV–hyperventilation; BH—breath holding; MBH—mild breath holding; ALT—Alanine Aminotransferase; higher than normal cholesterol or ALT levels are indicated in grey.

In addition, we subsequently investigated whether some miRNA pairs and/or classifiers were able to detect the various pathological processes associated with RTT. Table 4 demonstrates that patients with seizures could be differentiated from patients with no seizures with up to 78% accuracy. Ten RTT patients with walking problems were effectively differentiated from 20 ambulatory RTT patients (Table 5). Though, at present, the cause and effect relationship between walking problems associated with RTT and muscle-specific pathology remains to be determined, the best numerator in this case was miR-206, which is highly enriched in muscle cells. Furthermore, although liver pathology is often present in RTT patients, the alanine transaminase (ALT) levels were higher than those of the AMCs in only six RTT patients. These patients were effectively separated from the other RTT patients by multiple miRNA pairs, with liver-specific miR-122 being found to be the best numerator (Table 6). Cholesterol, the metabolic perturbations of which are characteristic of some patients with MeCP2 mutations, was also measured in all 30 RTT patients. Six and three patients had higher and lower than normal cholesterol levels, respectively (Table 3). Table 7 demonstrates that six patients with higher than normal cholesterol levels were differentiated from the other RTT patients with up to 82% accuracy.

**Table 4:** Differentiation of the RTT patients with seizures vs. RTT patients without seizures.

Seizures (12) / No seizures (18)			
miRNA pairs	Accuracy	AUC	P-value
**miR-132 / miR-491-5p**	0.78	0.85	2.80E-03
**miR-122 / miR-491-5p**	0.73	0.84	2.80E-03
**miR-122 / miR-29b-3p**	0.70	0.83	4.60E-03
**miR-122 / miR-146a**	0.71	0.83	5.90E-03
**miR-122 / miR-107**	0.72	0.81	5.90E-03
**miR-122 / miR-335-5p**	0.68	0.81	8.40E-03
**miR-122 / miR-155**	0.71	0.81	8.40E-03
**miR-16 / miR-491-5p**	0.69	0.81	9.40E-03
**miR-16 / miR-29b-3p**	0.69	0.81	5.90E-03
**let-7b / miR-491-5p**	0.70	0.81	7.50E-03
**miR-122 / miR-181a-5p**	0.66	0.80	8.40E-03

**Table 5:** Differentiation of the RTT patients with walking problems vs. ambulatory RTT patients.

Walking problems: WCB/NA (10) / AMB (20)			
miRNA pairs	Accuracy	AUC	P-value
**miR-206 / miR-125b**	0.79	0.95	1.70E-04
**miR-206 / miR-491-5p**	0.69	0.82	7.30E-03
**miR-206 / miR-29b-3p**	0.69	0.82	6.40E-03
**miR-206 / miR-107**	0.68	0.81	7.30E-03
**miR-206 / miR-132**	0.78	0.88	1.10E-03
**miR-206 / miR-335-5p**	0.68	0.81	7.30E-03
**miR-206 / miR-155**	0.70	0.84	5.00E-03
**miR-206 / let-7b**	0.73	0.83	6.40E-03
**miR-206 / miR-146a**	0.67	0.79	1.80E-02
**miR-206 / miR-134**	0.63	0.82	1.80E-02
**miR-206 / miR-181a-5p**	0.69	0.81	1.60E-02
**miR-206 / miR-409-3p**	0.65	0.80	1.50E-02
**miR-206 / miR-16**	0.74	0.83	5.70E-03
**miR-206 / miR-323-3p**	0.68	0.80	1.50E-02
**miR-206 / miR-411-5p**	0.65	0.78	2.80E-02
**miR-433-3p / miR-125b**	0.65	0.78	3.40E-02
**miR-432-5p / miR-411-5p**	0.65	0.75	4.90E-02

**Table 6:** Differentiation of the RTT patients with elevated ALT levels vs. RTT patients with normal ALT levels.

ALT-enzyme level: Elevated (6) / Normal (24)			
miRNA pairs	Accuracy	AUC	P-value
**miR-206 / miR-155**	0.82	0.94	8.50E-04
**miR-206 / miR-132**	0.81	0.94	1.40E-03
**miR-206 / miR-335-5p**	0.80	0.93	1.70E-03
**miR-122 / miR-335-5p**	0.74	0.92	2.80E-03
**miR-122 / miR-125b**	0.67	0.92	4.40E-03
**miR-206 / miR-146a**	0.80	0.92	2.00E-03
**miR-206 / miR-491-5p**	0.81	0.92	1.70E-03
**miR-206 / miR-107**	0.80	0.92	2.00E-03
**miR-206 / miR-29b-3p**	0.80	0.92	2.00E-03
**miR-122 / miR-155**	0.71	0.91	3.80E-03
**miR-206 / miR-181a-5p**	0.81	0.91	2.00E-03
**miR-122 / miR-146a**	0.69	0.90	3.20E-03
**miR-122 / miR-107**	0.78	0.90	4.40E-03
**miR-122 / miR-29b-3p**	0.74	0.90	3.80E-03
**miR-122 / miR-181a-5p**	0.74	0.90	5.10E-03
**miR-206 / miR-125b**	0.80	0.90	2.40E-03
**miR-122 / miR-132**	0.74	0.89	9.20E-03
**miR-122 / miR-491-5p**	0.73	0.88	6.90E-03
**miR-206 / miR-134**	0.72	0.88	8.00E-03
**miR-433-3p / miR-181a-5p**	0.73	0.88	5.10E-03
**miR-16 / miR-155**	0.75	0.87	9.20E-03
**miR-122 / let-7b**	0.64	0.86	1.60E-02
**miR-206 / let-7b**	0.70	0.86	1.10E-02
**miR-206 / miR-409-3p**	0.73	0.86	6.90E-03
**miR-206 / miR-323-3p**	0.74	0.86	8.00E-03
**miR-433-3p / miR-29b-3p**	0.74	0.86	1.40E-02
**miR-16 / miR-335-5p**	0.80	0.86	1.20E-02
**miR-16 / miR-132**	0.73	0.86	1.40E-02
**miR-433-3p / miR-146a**	0.72	0.85	9.20E-03

**Table 7:** Differentiation of the RTT patients with elevated cholesterol levels vs. RTT patients with normal cholesterol levels.

Cholesterol level: Elevated (6) / Normal (24)			
miRNA pairs	Accuracy	AUC	P-value
**miR-146a / miR-29b-3p**	0.82	0.92	1.20E-03
**miR-206 / miR-122**	0.75	0.89	6.00E-03
**miR-181a-5p / miR-409-3p**	0.56	0.86	4.10E-02
**miR-335-5p / miR-29b-3p**	0.69	0.85	1.40E-02
**miR-335-5p / let-7b**	0.73	0.84	1.60E-02
**miR-146a / miR-411-5p**	0.70	0.83	2.30E-02
**miR-107 / miR-29b-3p**	0.70	0.83	2.00E-02
**miR-155 / miR-125b**	0.58	0.83	2.00E-02
**miR-491-5p / miR-411-5p**	0.60	0.83	3.30E-02
**miR-146a / miR-432-5p**	0.68	0.82	3.30E-02
**miR-146a / miR-125b**	0.66	0.81	4.10E-02
**miR-146a / miR-323-3p**	0.62	0.81	3.70E-02
**miR-146a / miR-433-3p**	0.70	0.81	3.70E-02
**miR-206 / miR-125b**	0.68	0.81	2.30E-02
**miR-335-5p / miR-125b**	0.70	0.81	2.90E-02
**miR-335-5p / miR-134**	0.69	0.81	3.70E-02
**miR-181a-5p / miR-411-5p**	0.55	0.81	3.70E-02
**miR-181a-5p / miR-134**	0.59	0.81	3.70E-02
**miR-181a-5p / miR-323-3p**	0.53	0.81	3.70E-02
**miR-491-5p / miR-432-5p**	0.64	0.81	3.70E-02
**miR-146a / miR-409-3p**	0.68	0.80	3.70E-02
**miR-146a / miR-134**	0.67	0.80	4.10E-02
**miR-491-5p / miR-409-3p**	0.64	0.80	4.60E-02

## Discussion

The goal of this study was to determine whether circulating cell-free miRNAs in plasma can be used to reliably detect RTT patients, monitor disease development, and evaluate the different aspects of this disease, such as liver and muscle pathology. This study initially implemented animal RTT models to distinguish between Wt and *Mecp2* mutant mice as a step towards examining the utility of this approach in monitoring disease progression and treatment response in preclinical RTT models. Loss of *MECP2* can alter the transcription of many genes, including those encoding specific brain miRNAs [24], such as miR-132, an inhibitor of *MECP2* expression [24, 45–47]. Altered transcription and secretion of brain miRNAs may, in turn, lead to changes in the concentrations of these miRNAs in plasma. In fact, it has previously been shown that analysis of plasma levels of miRNAs enriched in neurites and synapses of brain regions affected by neurodegeneration yields effective miRNA biomarkers for detection and monitoring of neurodegenerative diseases compared to healthy controls [20, 21, 23]. Highly conserved miRNA biomarkers are advantageous because the same assay can be applied to both animal models and humans. Since plasma concentrations of brain-enriched miRNAs are relatively low, miRNA array and next-generation sequencing (NGS) approaches are not sufficiently sensitive for reliable detection of these miRNAs [23, 48], whereas the approach used in this study is based on targeted analysis of miRNAs by individual RT-qPCR. An additional challenge is that only a small volume of plasma (approximately 0.2 ml), which is sufficient to analyze only eight miRNAs by RT-qPCR, can be obtained from each mouse. Furthermore, changes in miRNA expression, not only in the brain but also in other organs, can affect plasma levels of ubiquitous miRNAs. Based on our previous data and analysis of the literature [23], in this study we tested seven brain-enriched miRNAs, as well as miR-16, a ubiquitous miRNA involved in apoptosis regulation, in four RTT murine models. In order to account for the potential effects of extraneous factors, such as plasma collection and specimen handling, samples from three different sites were tested. The results supported the hypothesis that miRNA pairs and their combinations, including several miRNA pairs that were uniformly effective biomarkers in all murine RTT models tested, are able to differentiate Null and Het mice from Wt mice with >90% accuracy.

In 2014, Cheng et al. [47] demonstrated that *MECP2* regulates biogenesis of some miRNAs and found changes in their expression, such as increases in miR-107 and miR-122 and decreases in miR-335-5p, miR-132 and miR-411 expression in the brain, due to *MECP2* mutations. These changes in miRNA expression may explain the effectiveness of certain miRNA pairs in our studies, for example miR-107/miR-335-5p, and miR-107/miR-132. On the other hand, although according to Cheng et al. expression of miR-323-3p increases, this miRNA proved to be a good denominator both in our animal and human studies. This discrepancy can be explained by various factors, including differences in genotypes and the ages of the animals tested in the two studies. Additional studies should be performed to address these questions.

Consistent with our hypothesis, individual miRNA pairs are more effective in Null (male) RTT models than in Het (female) models, indicating that disease severity correlates with miRNA ratios, with the only exception being miRNA pairs with miR-132 as the numerator (miR-132/miR-335-5p and miR-132/miR-323-3p; Table 2), which were equally effective in the Null and Het models.

We then extended our investigation to human patients, and the data obtained both supported and expanded the results of the mouse models studies due to the testing of more miRNAs, the analysis of RTT patients at several stages of pathological development, and the comparison of patients with different RTT symptoms. The most important result was that the same brain-enriched miRNAs formed biomarker pairs that could be used to detect RTT in both animal models and humans (Table 2). In addition, the human data demonstrated the applicability of our approach to the detection of liver and muscle pathologies and of changes in cholesterol metabolism. The analysis of various age groups indicated the importance of using AMCs for data analysis. The results generated in this study are preliminary due to the relatively small number of study participants, and larger studies are needed to select the best miRNA biomarkers; however, the similarity between the data obtained in the 4 animal models and those obtained in the human patients is highly promising.

In summary, the data presented here demonstrated that RTT biomarker development based on analysis of plasma miRNAs is feasible. Moreover, our finding that miRNA biomarker candidates more effectively differentiated male rather than female *Mecp2* mutants from controls in the mouse models studies, as well as in the human studies, suggested that these markers correlate with disease severity. This result, in turn, suggested that miRNA pairs have the potential to be developed as prognostic biomarkers for RTT and as markers of disease progression and treatment response. Finally, our data suggested that this approach could also prove fruitful for other neurodevelopmental disorders.

## Methods

### Animal models and plasma collection

Two mouse models of RTT, *Mecp2*^*tm1*.*1Jae*^ (deletion of exon 3)^14^ and *Mecp2*^*tm1*.*1Bird*^ (deletion of exons 3 and 4)^15^, were tested. Similar to RTT patients, these animals appear normal at birth and then develop RTT-like symptoms, including reduced brain size and weight, hypoactivity, abnormalities in locomotion (altered gait, hindlimb clasping), anxiety, cognitive deficits, and occasional hard respiration. *MECP2* is located on the X chromosome, and the severity of RTT depends on the type of mutation (*MECP2* can be completely or partially inactivated) and variability in X chromosome inactivation. The disease is much more severe in males. In the *Mecp2*^*tm1*.*1Bird*^ Null male model, mice appear normal until postnatal week 3, with symptoms progressing between postnatal weeks 3 and 5, leading to death around postnatal week 8. *Mecp2*^*tm1*.*1Bird*^ Het females are asymptomatic up to postnatal week 6 when initial symptoms appear, and animals die by 11–12 months of life. In *Mecp2*^*tm1*.*1Jae*^ Null males, progression of symptoms occurs between postnatal weeks 5 and 8 leading to death around 2.5 months of life. In *Mecp2*^*tm1*.*1Jae*^ Het females, symptoms appear by postnatal week 15, and animals die at 12 months of life.

Male (Null) and female (Het) *Mecp2*^*tm1*.*1Jae*^ (Case Western) mice and *Mecp2*^*tm1*.*1Bird*^ (Jackson Labs, Psychogenics) mice were used and compared to wild-type (Wt) mice as follows:

*Mecp2*^*tm1*.*1Jae*^: (i) 11 Null vs 9 Wt (5–6 weeks old) and (ii) 6 Het vs 7 Wt (6 months old)

*Mecp2*^*tm1*.*1Bird*^: (iii) 8 Null vs 10 Wt (5–6 weeks old) and (iv) 10 Het vs 10 Wt (18 weeks old)

Mice were anesthetized with isoflurane gas. Whole blood was collected into K2EDTA tubes via cardiac puncture. Samples were centrifuged at 2000 g for 15 minutes at 4°C, and approximately 0.2 ml of plasma was transferred to Eppendorf tubes and frozen on dry ice. All animal studies were conducted in accordance with the relevant guidelines and regulations and were approved by the corresponding IACUCs of Case Western Reserve University School of Medicine, Jackson Labs, and Psychogenics.

### Study participants and plasma collection

All patients with RTT were recruited from the Tri State Rett Syndrome Center (NY) database. The parents were informed about the study prior to their scheduled annual health care maintenance visit, and the patients were recruited during the visit. Written, informed consent was provided by all patients and/or their legal guardians. The study protocol and procedures were approved by the Institutional Review Board of Albert Einstein College of Medicine, Montefiore Medical Center and were conducted in accordance with the Declaration of Helsinki.

All study participants with RTT were experiencing their usual, baseline health at the time of their recruitment and of the blood draw. Control study participants were recruited from the pediatric waiting area at the Children's Hospital at Montefiore from among healthy siblings (with no neurological developmental problems and who were not on medications), who were accompanying their brothers/sisters to their medical appointments. Control study participants were age- and gender-matched to the recruited patients with RTT. The clinical information regarding the study participants is summarized in Table 3. Five high functioning (HF) patients were excluded from the analysis due to the small sample size and wide age distribution.

Whole blood from 30 RTT patients and 30 AMCs was collected via venipuncture into K2EDTA vacutainers, and the plasma was separated as described above and frozen at -70°C.

### RNA isolation and RT-qPCR

In each experiment, RNA was extracted from 200 μl of plasma (two preps obtained from the same human study participant were then combined) using a TRIzol treatment and silica (Ambiom Glass Fiber Microcolumn) binding protocol (http://asuragen.com/wp-content/uploads/2016/05/biomarkers.pdf). Single-target quantitative RT-PCR (RT-qPCR) was performed using the TaqMan Reverse Transcription Kit and miRNA-specific stem-loop primers (ThermoFisher). The RT step was performed in triplicate, and 2 μl of the original plasma equivalents was present in the final PCR. Placental RNA was used as a “positive control”, and no-template controls were used as a “negative control” for each run. Calibration curves were obtained for each miRNA tested. Quality control of the miRNA preps was performed by testing two ubiquitous miRNAs, namely, miR-16 and miR-27a, known to have low variability in each plasma preparation.

### Statistical methods

All Ct values were recalculated for 1 μl of plasma based on the calibration curves. DiamiR’s custom software (20) was used for all calculations, including the identification of miRNA pairs capable of differentiating control and mutant *Mecp2* mice based on their concentration ratios (ΔCt) in plasma. Mann-Whitney U tests were used to evaluate the significance of the differences between any two experimental groups based on the various biomarker miRNA pairs. Receiver operating characteristic (ROC) curves were constructed, and the AUC values were calculated to evaluate the sensitivity and specificity of the miRNA pairs. The sensitivity and specificity are reported for the cutoff points on the ROC curves that provided the best overall accuracy.

## Supporting information

S1 Table RTT and AMC groups: Number of participants and age ranges (average ± standard deviation). (TIF)

S2 Table A) miRNA pairs that differentiate AMC groups do not distinguish the corresponding RTT age groups. B) miRNA pairs that differentiate RTT age groups do not distinguish the corresponding AMC groups.
(PDF)

S1 Fig Differentiation between Mutant and Control male mice genotypes. **A)** Mutant Genotype: *Mecp2*^*tm1*.*1Jae*^ Null; CNTR–Control. **B)** Mutant Genotype: *Mecp2*^*tm1*.*1Bird*^ Null; CNTR–Control.
(PDF)

S2 Fig Differentiation between Mutant and Control female mice genotypes. **A)** Mutant Genotype: *Mecp2*^*tm1*.*1Jae*^ Null; CNTR–Control. **B)** Mutant Genotype: *Mecp2*^*tm1*.*1Bird*^ Null; CNTR–Control.
(PDF)

S3 Fig Differentiation of RTT age groups from each other by miRNA pairs and their combinations. (PDF)
