# Supplementary figures and images for: Brain-enriched microRNAs circulating in plasma as novel biomarkers for Rett syndrome
Source: PLoS One. 2019 Jul 10;14(7):e0218623. doi: 10.1371/journal.pone.0218623 (PMC6619658; doi:10.1371/journal.pone.0218623)

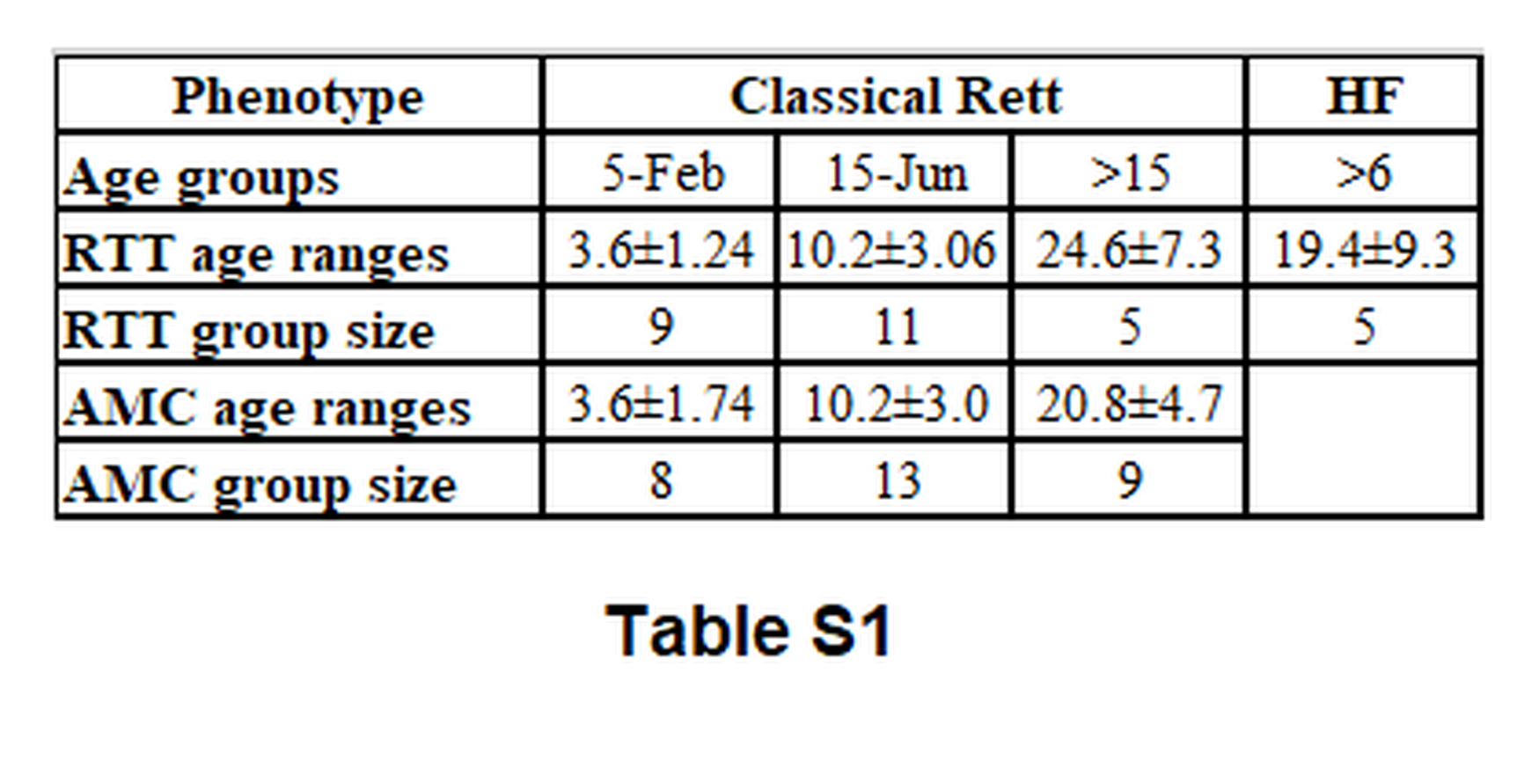

Supplement: S1 Table — (TIF) [file pone.0218623.s001.tif]
