# Supplementary material for: Brain-enriched microRNAs circulating in plasma as novel biomarkers for Rett syndrome
Source: PLoS One. 2019 Jul 10;14(7):e0218623. doi: 10.1371/journal.pone.0218623 (PMC6619658; doi:10.1371/journal.pone.0218623)
Supplement: S2 Table — A) miRNA pairs that differentiate AMC groups do not distinguish the corresponding RTT age groups. B) miRNA pairs that differentiate RTT age groups do not distinguish the corresponding AMC groups. (PDF) [file pone.0218623.s002.pdf]

Participants	miRNA pairs	CNTR					RTT				
		Sens	Spec	Accur	AUC	P-value	Sens	Spec	Accur	AUC	P-value
CNTR 6-15 y.o.(13) / CNTR 2-5 y.o.(8)	miR-146a / miR-125b	0.89	0.85	0.86	0.9	3.10E-03	0.8	0.45	0.56	0.52	2.80E-01
	miR-432-5p / miR-125b	0.89	0.77	0.82	0.86	8.10E-03	0.57	0.6	0.59	0.68	2.50E-01
	miR-107 / miR-125b	0.8	0.79	0.8	0.89	3.80E-03	0.67	0.3	0.42	0.54	1.50E-01
	miR-335-5p / miR-125b	0.9	0.7	0.78	0.88	5.60E-03	0.8	0.45	0.56	0.68	2.50E-01
	miR-411-5p / miR-323-3p	0.78	0.77	0.77	0.9	1.60E-03	0.26	0.48	0.41	0.46	2.10E-01
	miR-206 / miR-125b	0.89	0.69	0.77	0.87	5.60E-03	0.8	0.73	0.75	0.81	8.60E-02
	miR-433-3p / miR-125b	0.69	0.8	0.76	0.87	9.70E-03	0.58	0.62	0.61	0.74	1.30E-01
	miR-134 / miR-125b	0.69	0.8	0.76	0.88	5.60E-03	0.64	0.48	0.53	0.71	2.10E-01
	miR-155 / miR-125b	0.82	0.73	0.76	0.89	4.60E-03	0.56	0.34	0.41	0.55	4.10E-01
	miR-411-5p / miR-125b	0.69	0.79	0.75	0.86	5.60E-03	0.8	0.55	0.62	0.74	1.50E-01
	miR-107 / miR-132-3p	0.69	0.79	0.75	0.87	4.60E-03	0.47	0.32	0.37	0.46	1.80E-01
	miR-29b-5p / miR-125b	0.78	0.69	0.73	0.87	9.70E-03	0.6	0.27	0.38	0.52	2.80E-01
	miR-146a / miR-132-3p	0.75	0.67	0.7	0.86	5.60E-03	0.51	0.47	0.48	0.59	4.10E-01
CNTR >15 y.o.(9) / CNTR 6-15 y.o.(13)	miR-335-5p / miR-134	0.92	0.88	0.9	0.99	1.20E-04	0.45	0.56	0.5	0.61	4.10E-01
	miR-146a / miR-134	0.83	0.74	0.79	0.91	1.70E-03	0.58	0.36	0.48	0.58	4.70E-01
	miR-491-5p / miR-134	0.8	0.78	0.79	0.94	6.20E-04	0.49	0.48	0.48	0.56	4.70E-01
	miR-107 / miR-134	0.85	0.63	0.76	0.94	1.00E-03	0.64	0.44	0.55	0.66	3.20E-01
	miR-107 / miR-409-3p	0.79	0.7	0.76	0.9	3.30E-03	0.53	0.64	0.58	0.68	2.50E-01
	miR-181a-5p / miR-134	0.85	0.63	0.76	0.91	1.70E-03	0.6	0.63	0.62	0.71	2.50E-01
	let-7b / miR-125b	0.81	0.66	0.75	0.87	7.50E-03	0.58	0.35	0.47	0.54	3.80E-01
	miR-335-5p / miR-409-3p	0.83	0.54	0.72	0.93	3.30E-03	0.39	0.71	0.54	0.62	3.80E-01
	miR-16 / miR-125b	0.65	0.83	0.72	0.87	7.50E-03	0.33	0.6	0.45	0.52	2.20E-01
	miR-335-5p / miR-323-3p	0.68	0.74	0.7	0.86	9.20E-03	0.43	0.64	0.53	0.63	3.00E-01

S2a Table

Participants	miRNA pairs	RTT					CNTR				
		Sens	Spec	Accur	AUC	P-value	Sens	Spec	Accur	AUC	P-value
RTT 6-15 y.o.(11)/2-5 y.o. pairs(9)	miR-122 / miR-155	0.91	0.89	0.9	0.95	9.10E-04	0.38	0.61	0.46	0.55	4.30E-01
	miR-122 / miR-335-5p	0.91	0.89	0.9	0.94	1.50E-03	0.36	0.46	0.4	0.45	1.60E-01
	miR-132-3p / miR-155	0.91	0.89	0.9	0.96	5.40E-04	0.43	0.59	0.49	0.55	3.70E-01
	miR-122 / miR-146a	0.82	0.89	0.85	0.92	3.10E-03	0.36	0.58	0.44	0.5	2.50E-01
	miR-122 / miR-491-5p	0.82	0.89	0.85	0.92	3.10E-03	0.32	0.64	0.44	0.51	2.90E-01
	miR-122 / miR-16	0.84	0.8	0.83	0.89	9.20E-03	0.46	0.38	0.43	0.55	3.70E-01
	miR-122 / miR-29b-5p	0.73	0.89	0.8	0.93	2.50E-03	0.31	0.63	0.43	0.49	2.20E-01
	miR-122 / miR-107	0.73	0.89	0.8	0.92	3.90E-03	0.31	0.63	0.43	0.49	2.50E-01
	miR-132-3p / miR-323-3p	0.82	0.78	0.8	0.89	7.50E-03	0.54	0.38	0.48	0.63	4.30E-01
	miR-122 / miR-432-5p	0.76	0.82	0.79	0.92	1.90E-03	0.44	0.48	0.45	0.54	4.30E-01
	miR-122 / miR-411-5p	0.76	0.82	0.79	0.92	2.50E-03	0.42	0.54	0.47	0.56	4.60E-01
	miR-132-3p / miR-432-5p	0.85	0.69	0.78	0.89	7.50E-03	0.44	0.48	0.45	0.56	4.00E-01
	miR-122 / miR-409-3p	0.73	0.78	0.75	0.88	6.10E-03	0.49	0.66	0.56	0.68	2.70E-01
	miR-181a-5p / miR-411-5p	0.73	0.78	0.75	0.86	2.00E-02	0.46	0.5	0.48	0.6	4.30E-01
	let-7b / miR-155	0.79	0.64	0.73	0.86	7.50E-03	0.56	0.52	0.55	0.69	2.00E-01
	miR-132-3p / miR-411-5p	0.67	0.81	0.73	0.86	1.10E-02	0.43	0.46	0.44	0.54	4.30E-01
	miR-323-3p / miR-432-5p	0.7	0.75	0.72	0.86	7.50E-03	0.33	0.54	0.41	0.48	2.50E-01
	let-7b / miR-432-5p	0.73	0.67	0.7	0.86	1.10E-02	0.57	0.46	0.53	0.6	4.30E-01
	miR-122 / miR-323-3p	0.58	0.83	0.69	0.9	3.90E-03	0.45	0.48	0.46	0.6	4.30E-01
	miR-122 / miR-433-3p	0.57	0.81	0.68	0.89	3.90E-03	0.43	0.46	0.44	0.56	4.60E-01
	miR-409-3p / miR-432-5p	0.52	0.85	0.67	0.85	1.10E-02	0.23	0.63	0.38	0.41	1.00E-01
	miR-181a-5p / miR-155	0.59	0.72	0.65	0.85	2.00E-02	0.62	0.63	0.62	0.66	2.70E-01
	miR-122 / miR-181a-5p	0.49	0.8	0.63	0.86	1.40E-02	0.38	0.5	0.43	0.52	2.50E-01
RTT >15 y.o.(5) /6-15 y.o. pairs(11)	miR-122 / miR-491-5p	0.8	0.91	0.87	0.94	6.10E-03	0.48	0.41	0.44	0.41	1.10E-01
	miR-122 / miR-181a-5p	0.8	0.91	0.87	0.93	8.40E-03	0.46	0.4	0.43	0.5	1.90E-01
	miR-122 / miR-29b-5p	0.83	0.85	0.84	0.93	8.40E-03	0.32	0.58	0.47	0.43	1.40E-01
	miR-335-5p / miR-491-5p	0.83	0.85	0.84	0.95	3.10E-03	0.44	0.46	0.45	0.56	4.20E-01
	miR-122 / miR-155	0.82	0.84	0.83	0.91	1.50E-02	0.34	0.55	0.46	0.48	2.50E-01
	miR-335-5p / miR-181a-5p	0.81	0.83	0.82	0.89	2.60E-02	0.53	0.52	0.52	0.63	2.70E-01
	miR-411-5p / miR-491-5p	0.8	0.82	0.81	0.88	2.60E-02	0.74	0.44	0.56	0.73	1.30E-01
	miR-122 / miR-107	0.85	0.77	0.8	0.92	1.10E-02	0.33	0.46	0.41	0.41	1.00E-01
	miR-433-3p / miR-491-5p	0.85	0.77	0.8	0.94	4.40E-03	0.51	0.42	0.45	0.53	3.70E-01
	miR-122 / miR-146a	0.63	0.85	0.78	0.89	1.50E-02	0.32	0.52	0.44	0.45	1.40E-01
	miR-155 / miR-491-5p	0.82	0.75	0.77	0.88	2.00E-02	0.29	0.4	0.35	0.45	4.70E-02
	miR-433-3p / miR-181a-5p	0.8	0.73	0.75	0.87	2.00E-02	0.73	0.58	0.64	0.74	1.30E-01
	miR-432-5p / miR-491-5p	0.8	0.73	0.75	0.87	2.60E-02	0.7	0.42	0.53	0.65	2.70E-01
	miR-335-5p / miR-146a	0.8	0.73	0.75	0.88	2.00E-02	0.56	0.62	0.59	0.75	1.40E-01
	let-7b / miR-491-5p	0.6	0.82	0.75	0.86	2.60E-02	0.33	0.38	0.36	0.39	5.40E-02
	miR-335-5p / miR-107	0.86	0.69	0.74	0.94	6.10E-03	0.49	0.34	0.4	0.5	2.50E-01
	miR-122 / miR-132-3p	0.42	0.86	0.72	0.88	1.50E-02	0.46	0.47	0.47	0.54	3.40E-01
	miR-146a / miR-491-5p	0.58	0.78	0.72	0.87	1.50E-02	0.36	0.5	0.44	0.51	3.40E-01
	miR-16 / miR-491-5p	0.85	0.58	0.67	0.85	3.40E-02	0.33	0.38	0.36	0.37	4.10E-02
	miR-433-3p / miR-107	0.67	0.61	0.63	0.86	4.40E-02	0.55	0.45	0.49	0.57	4.70E-01

S2b Table
