# Supplementary material for: Brain-enriched microRNAs circulating in plasma as novel biomarkers for Rett syndrome
Source: PLoS One. 2019 Jul 10;14(7):e0218623. doi: 10.1371/journal.pone.0218623 (PMC6619658; doi:10.1371/journal.pone.0218623)
Supplement: S1 Fig — A) Mutant Genotype: Mecp2tm1.1Jae Null; CNTR–Control. B) Mutant Genotype: Mecp2tm1.1Bird Null; CNTR–Control. (PDF) [file pone.0218623.s003.pdf]

| Genotype | Pairs | Sens | Spec | Accur | AUC | P-value |
|---|---|---|---|---|---|---|
| *Mecp2 / tm1. 1Jae* | miR-107 / miR-370 | 0.74 | 0.79 | 0.76 | 0.93 | 1.90E-03 |
| | miR-107 / miR-323-3p | 0.73 | 1 | 0.84 | 0.98 | 4.00E-04 |
| | miR-107 / miR-335-5p | 0.91 | 0.75 | 0.84 | 0.96 | 5.40E-04 |
| | miR-107 / miR-411-5p | 0.82 | 0.75 | 0.79 | 0.93 | 1.70E-03 |
| | miR-107 / miR-132-3p | 0.91 | 0.88 | 0.89 | 0.95 | 1.30E-03 |
| | miR-107 / miR-16 | 0.76 | 0.65 | 0.71 | 0.87 | 5.80E-03 |
| | miR-491-5p / miR-370 | 0.74 | 0.8 | 0.77 | 0.93 | 2.50E-03 |
| | miR-491-5p / miR-323-3p | 0.82 | 0.88 | 0.84 | 0.98 | 4.00E-04 |
| | miR-491-5p / miR-335-5p | 0.91 | 0.88 | 0.89 | 0.95 | 9.60E-04 |
| | miR-491-5p / miR-411-5p | 0.66 | 0.77 | 0.71 | 0.88 | 7.30E-03 |
| | miR-491-5p / miR-132-3p | 0.91 | 0.88 | 0.89 | 0.93 | 2.80E-03 |
| | miR-491-5p / miR-16 | 0.91 | 0.88 | 0.89 | 0.97 | 4.00E-04 |
| | miR-16 / miR-323-3p | 0.78 | 0.72 | 0.76 | 0.9 | 3.60E-03 |
| | miR-16 / miR-335-5p | 0.74 | 0.79 | 0.76 | 0.89 | 7.30E-03 |
| | miR-132-3p / miR-370 | 0.42 | 0.79 | 0.59 | 0.85 | 4.10E-02 |
| | miR-132-3p / miR-335-5p | 0.63 | 0.76 | 0.68 | 0.86 | 1.70E-02 |
| | miR-411-5p / miR-370 | 0.78 | 0.73 | 0.76 | 0.89 | 5.60E-03 |
| | miR-107 / miR-132-3p + miR-491-5p / miR-16 + miR-411-5p / miR-370 | 1 | 1 | 1 | 1 | 1.20E-04 |

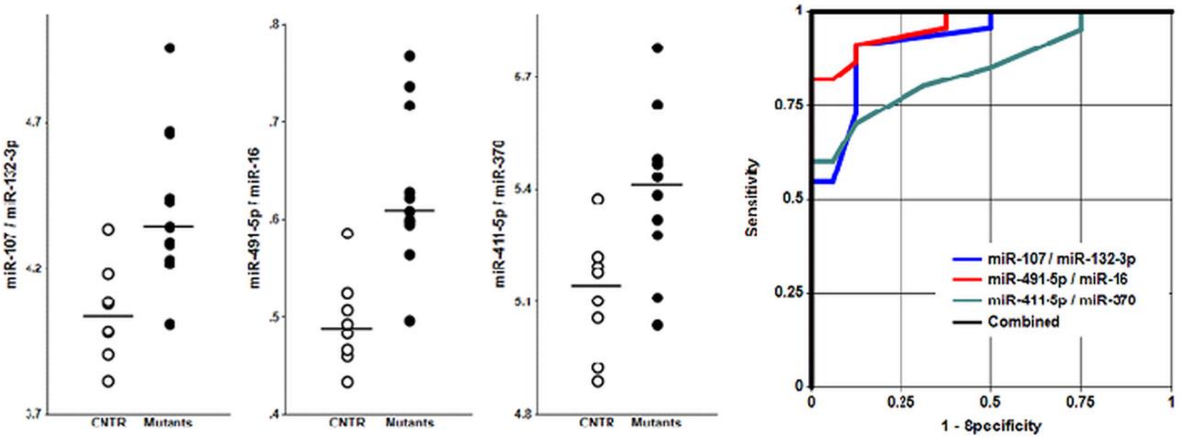


**S1a Fig.**

| Genotype | Pairs | Sens | Spec | Accur | AUC | P-value |
|---|---|---|---|---|---|---|
| ***Mecp2 / tm1.1Bird*** | **miR-411-5p / miR-335-5p** | 0.89 | 0.91 | 0.9 | 0.98 | 5.70E-04 |
| | **miR-411-5p / miR-132-3p** | 0.88 | 0.81 | 0.84 | 0.93 | 3.30E-03 |
| | **miR-107 / miR-335-5p** | 1 | 0.9 | 0.94 | 0.99 | 2.20E-04 |
| | **miR-107 / miR-132-3p** | 1 | 0.8 | 0.89 | 0.98 | 4.20E-04 |
| | **miR-491-5p / miR-335-5p** | 0.88 | 1 | 0.94 | 0.98 | 5.70E-04 |
| | **miR-491-5p / miR-132-3p** | 1 | 0.9 | 0.94 | 0.99 | 2.20E-04 |
| | **miR-16 / miR-335-5p** | 0.89 | 0.91 | 0.9 | 0.98 | 5.70E-04 |
| | **miR-16 / miR-132-3p** | 1 | 1 | 1 | 1 | 1.50E-04 |
| | **miR-323-3p / miR-335-5p** | 0.88 | 0.9 | 0.89 | 0.98 | 4.20E-04 |
| | **miR-323-3p / miR-132-3p** | 0.89 | 0.72 | 0.79 | 0.91 | 7.10E-03 |
| | **miR-107 / miR-132-3p +<br>miR-491-5p / miR-132-3p +<br>miR-323-3p / miR-132-3p** | 1 | 1 | 1 | 1 | 2.20E-04 |

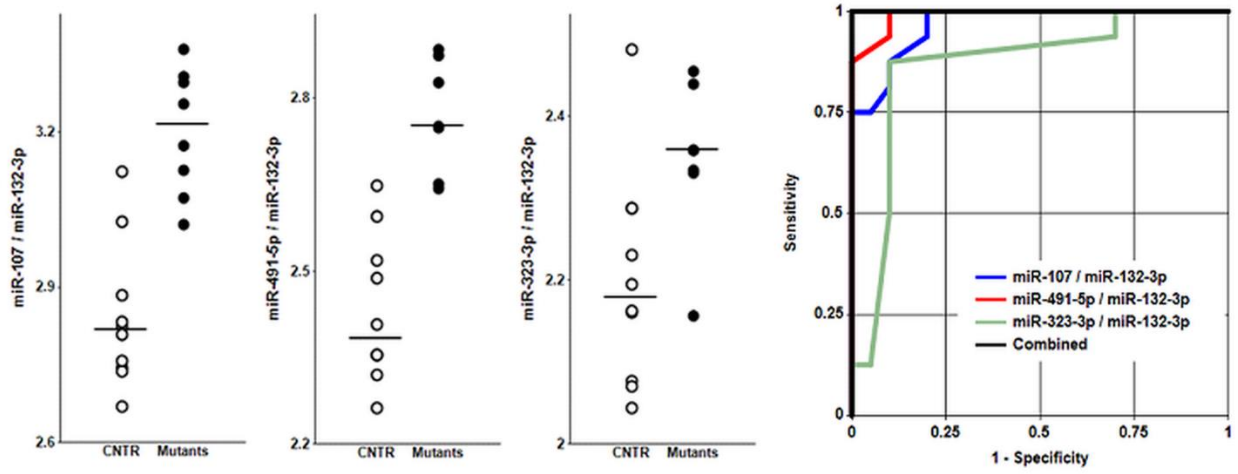


**S1b Fig.**
