# Supplementary material for: Brain-enriched microRNAs circulating in plasma as novel biomarkers for Rett syndrome
Source: PLoS One. 2019 Jul 10;14(7):e0218623. doi: 10.1371/journal.pone.0218623 (PMC6619658; doi:10.1371/journal.pone.0218623)
Supplement: S2 Fig — A) Mutant Genotype: Mecp2tm1.1Jae Null; CNTR–Control. B) Mutant Genotype: Mecp2tm1.1Bird Null; CNTR–Control. (PDF) [file pone.0218623.s004.pdf]

Genotype	Pairs	Sens	Spec	Accur	AUC	P-value
Mecp2 / tm1.1Jae	miR-107 / miR-335-5p	0.77	0.66	0.71	0.93	1.30E-02
	miR-107 / miR-491-5p	0.65	0.69	0.67	0.8	8.70E-02
	miR-107 / miR-323-3p	0.63	0.54	0.58	0.79	2.20E-01
	miR-16 / miR-335-5p	0.52	0.89	0.72	0.95	6.20E-03
	miR-16 / miR-491-5p	0.67	0.71	0.69	0.83	5.00E-02
	miR-16 / mir-411-5p	0.71	0.61	0.66	0.79	1.40E-01
	miR-132-3p / miR-335-5p	0.85	0.73	0.79	0.96	4.10E-03
	miR-132-3p / miR-491-5p	0.67	0.57	0.62	0.86	8.70E-02
	miR-132-3p / miR-323-3p	0.62	0.8	0.71	0.81	1.10E-01
	Let-7e / miR-335-5p	0.79	0.54	0.65	0.85	3.70E-02
	miR-491-5p / miR-335-5p	0.7	0.45	0.57	0.79	1.40E-01
	miR-16 / mir-411-5p + Let-7e / miR-335-5p + miR-132-3p / miR-335-5p	1	1	1	1	1.10E-03

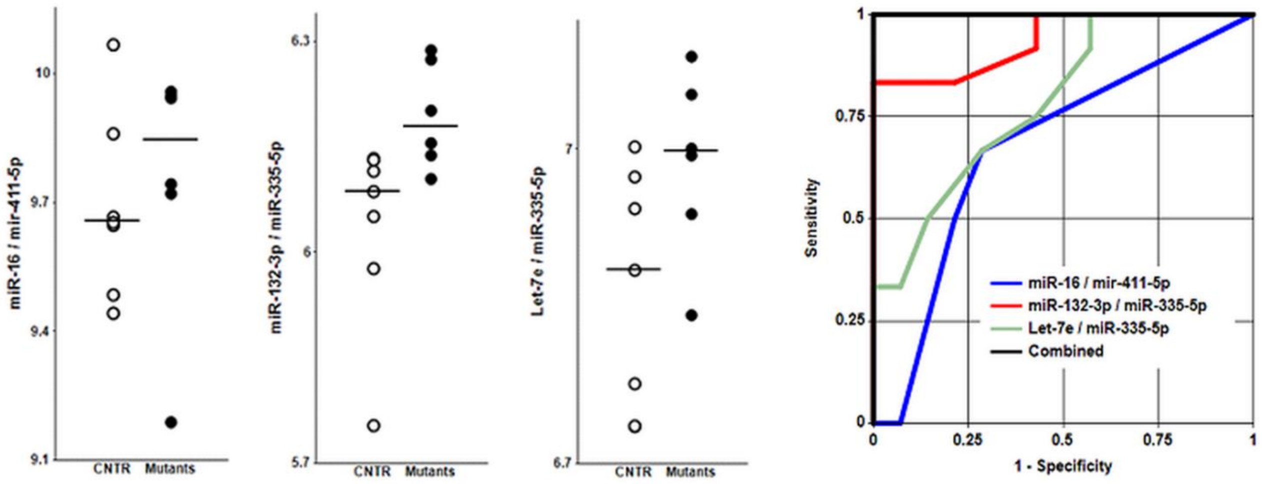

S2a Fig.

Genotype	Pairs	Sens	Spec	Accur	AUC	P-value
Mecp2 / tm1.1Bird	miR-107 / miR-335-5p	0.49	0.78	0.63	0.8	3.20E-02
	miR-107 / miR-132-3p	0.54	0.76	0.65	0.82	2.30E-02
	miR-491-5p / miR-335-5p	0.73	0.52	0.63	0.78	4.40E-02
	miR-491-5p / miR-323-3p	0.57	0.75	0.66	0.77	5.20E-02
	miR-491-5p / miR-132-3p	0.51	0.71	0.61	0.82	4.40E-02
	miR-16 / miR-335-5p	0.63	0.63	0.63	0.75	1.10E-01
	miR-16 / miR-323-3p	0.59	0.69	0.64	0.75	7.00E-02
	miR-107 / miR-132-3p + miR-491-5p / miR-335-5p + miR-491-5p / miR-323-3p	0.9	0.7	0.8	0.83	3.70E-02

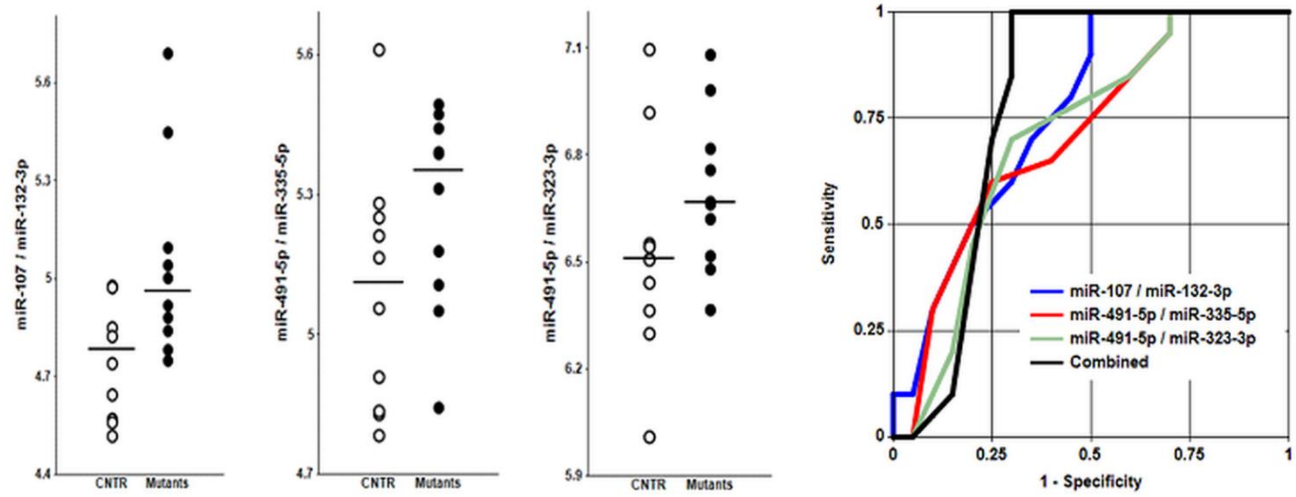

S2b Fig.
