# Supplementary material for: Brain-enriched microRNAs circulating in plasma as novel biomarkers for Rett syndrome
Source: PLoS One. 2019 Jul 10;14(7):e0218623. doi: 10.1371/journal.pone.0218623 (PMC6619658; doi:10.1371/journal.pone.0218623)
Supplement: S3 Fig — (PDF) [file pone.0218623.s005.pdf]

Participants	miRNA pairs	Sens	Spec	Accur	AUC	P-value
RTT 6-15 y.o.(11) / RTT 2-5 y.o.(9)	miR-132-3p / miR-155	0.91	0.89	0.9	0.96	5.40E-04
	miR-122 / miR-155	0.91	0.89	0.9	0.95	9.10E-04
	miR-122 / miR-335-5p	0.91	0.89	0.9	0.94	1.50E-03
	miR-122 / miR-29b-3p	0.73	0.89	0.8	0.93	2.50E-03
	miR-122 / miR-433-3p	0.57	0.81	0.68	0.89	3.90E-03
	miR-122 / miR-16	0.84	0.8	0.83	0.89	9.20E-03
	miR-132-3p / miR-432-5p	0.85	0.69	0.78	0.89	7.50E-03
	miR-132-3p / miR-323-3p	0.82	0.78	0.8	0.89	7.50E-03
	miR-122 / miR-409-3p	0.73	0.78	0.75	0.88	6.10E-03
	miR-122 / miR-181a-5p	0.49	0.8	0.63	0.86	1.40E-02
	miR-181a-5p / miR-411-5p	0.73	0.78	0.75	0.86	2.00E-02
	miR-323-3p / miR-432-5p	0.7	0.75	0.72	0.86	7.50E-03
	miR-181a-5p / miR-155	0.59	0.72	0.65	0.85	2.00E-02
	miR-409-3p / miR-432-5p	0.52	0.85	0.67	0.85	1.10E-02
	miR-132-3p / miR-155 + miR-122 / miR-16 + miR-181a-5p / miR-411-5p	1	1	1	1	7.20E-05
	miR-132-3p / miR-155 + miR-181a-5p / miR-411-5p + miR-323-3p / miR-432-5p	1	1	1	1	7.20E-05
RTT >15 y.o.(5) / RTT 6-15 y.o.(11)	miR-335-5p / miR-491-5p	0.83	0.85	0.84	0.95	3.10E-03
	miR-122 / miR-491-5p	0.8	0.91	0.87	0.94	6.10E-03
	miR-433-3p / miR-491-5p	0.85	0.77	0.8	0.94	4.40E-03
	miR-335-5p / miR-107	0.86	0.69	0.74	0.94	6.10E-03
	miR-122 / miR-181a-5p	0.8	0.91	0.87	0.93	8.40E-03
	miR-122 / miR-29b-3p	0.83	0.85	0.84	0.93	8.40E-03
	miR-122 / miR-107	0.85	0.77	0.8	0.92	1.10E-02
	miR-122 / miR-155	0.82	0.84	0.83	0.91	1.50E-02
	miR-122 / miR-146a	0.63	0.85	0.78	0.89	1.50E-02
	miR-335-5p / miR-181a-5p	0.81	0.83	0.82	0.89	2.60E-02
	miR-122 / miR-132-3p	0.42	0.86	0.72	0.88	1.50E-02
	miR-411-5p / miR-491-5p	0.8	0.82	0.81	0.88	2.60E-02
	miR-335-5p / miR-146a	0.8	0.73	0.75	0.88	2.00E-02
	miR-155 / miR-491-5p	0.82	0.75	0.77	0.88	2.00E-02
	miR-433-3p / miR-181a-5p	0.8	0.73	0.75	0.87	2.00E-02
	miR-432-5p / miR-491-5p	0.8	0.73	0.75	0.87	2.60E-02
	miR-146a / miR-491-5p	0.58	0.78	0.72	0.87	1.50E-02
	miR-335-5p / miR-491-5p + miR-335-5p / miR-107 + miR-433-3p / miR-181a-5p	1	1	1	1	7.10E-04
	miR-335-5p / miR-181a-5p + miR-146a / miR-491-5p + miR-432-5p / miR-491-5p	1	1	1	1	7.10E-04
	miR-335-5p / miR-146a + miR-146a / miR-491-5p + miR-432-5p / miR-491-5p	1	1	1	1	7.10E-04
RTT >15 y.o.(5) / RTT 2-5 y.o.(9)	miR-122 / miR-491-5p	1	0.89	0.93	0.99	1.60E-03
	miR-122 / miR-155	1	0.89	0.93	0.99	1.60E-03
	miR-122 / miR-29b-3p	1	0.89	0.93	0.99	1.60E-03
	miR-122 / miR-125b	1	0.89	0.93	0.99	1.60E-03
	miR-122 / miR-107	1	0.89	0.93	0.98	2.50E-03
	miR-122 / miR-146a	1	0.89	0.93	0.97	3.80E-03
	miR-122 / miR-132-3p	1	0.89	0.93	0.97	3.80E-03
	miR-335-5p / miR-491-5p	0.88	0.73	0.79	0.97	3.80E-03
	miR-122 / miR-181a-5p	1	0.89	0.93	0.94	8.10E-03
	miR-122 / miR-409-3p	0.8	0.89	0.86	0.94	5.60E-03
	miR-122 / miR-335-5p	1	0.89	0.93	0.94	8.10E-03
	miR-122 / miR-433-3p	0.85	0.83	0.84	0.94	5.60E-03
	miR-122 / miR-323-3p	0.85	0.83	0.84	0.94	5.60E-03
	let-7b / miR-155	0.8	0.89	0.86	0.94	5.60E-03
	miR-122 / miR-411-5p	0.85	0.83	0.84	0.93	8.10E-03
	miR-206 / miR-181a-5p	0.6	0.78	0.71	0.88	3.10E-02
	miR-206 / miR-146a	0.8	0.78	0.79	0.88	2.30E-02
	miR-206 / miR-107	0.83	0.81	0.82	0.93	1.60E-02
	miR-16 / miR-107	0.85	0.59	0.68	0.88	4.10E-02
	miR-335-5p / miR-155	0.84	0.7	0.75	0.88	2.30E-02
	miR-122 / miR-335-5p + miR-335-5p / miR-155 + miR-206 / miR-107	1	1	1	1	1.10E-03

Figure S3.

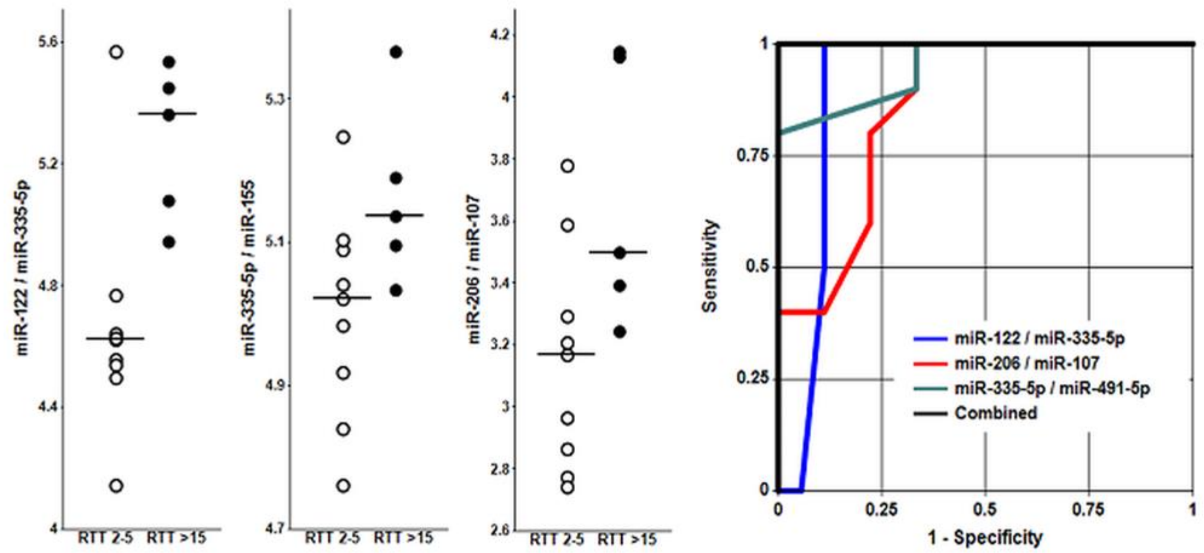

Figure S3 (cont.).
